# Morphea “en coup de sabre”: An unusual oral presentation

**DOI:** 10.4317/jced.53151

**Published:** 2017-02-01

**Authors:** Sven Niklander, Constanza Marín, René Martínez, Alfredo Esguep

**Affiliations:** 1 DDS, MDent, MSc, Department of Oral Medicine and Oral Pathology, Universidad Andres Bello, Viña del Mar, Chile. Avenida Valparaíso 1560, Viña del Mar, Chile; 2 DDS, Department of Oral Medicine and Oral Pathology, Universidad Andres Bello, Viña del Mar, Chile. Avenida Valparaíso 1560, Viña del Mar, Chile; 3 DDS, MSc, Department of Oral Medicine and Oral Pathology, Universidad Andres Bello, Viña del Mar, Chile. Avenida Valparaíso 1560, Viña del Mar, Chile

## Abstract

Morphea, or localized scleroderma, is an inflammatory disease that leads to sclerosis of the skin and underlying tissues due to excessive collagen deposition. Oral involvement is unusual and it may produce white linear fibrotic areas with a scar-like appearance, atrophy of tongue papillae, gingival recession and alveolar bone resorption. We report a case of a 13-year-old girl who consulted for progressive recession on the attached gingiva of her upper left incisors. She also presented a hypopigmented line on the left side skin of her upper lip, which continued through the vermilion and the lip mucosa, including the gingiva of the affected teeth. Clinical examination, blood tests, computerized axial tomography, echo-Doppler ultrasound and histopathological evaluation confirmed the diagnosis of morphea. Treatment with methotrexate and systemic corticosteroids was conducted. After 24 months, no other lesions appeared. No adverse side effects have been reported so far.

** Key words:**Localized scleroderma, oral morphea, linear scleroderma, oral involvement, intraoral lesions.

## Introduction

Morphea, or localized scleroderma, is an inflammatory disease that leads to sclerosis of the skin and underlying tissues due to excessive collagen deposition. It differs from systemic scleroderma due to the absence of Raynaud’s phenomenon and the compromise of organs such as the heart, lung, kidneys and gastrointestinal system (1,2). Its incidence ranges from 0.4 to 2.7 cases per 100,000 individuals/year, and is more common in Caucasian women, with a female:male ratio of 2.4-4.2:1.

The classification of the Pediatric Rheumatology European Society (3) recognizes five main types of which linear scleroderma is presented as a unilateral lesion that affects the extremities, face or scalp. In mild cases, only a linear area of ivory-colored pigmentation can be observed on the surface of the skin; however, deeper compromise may affect the subcutaneous tissue, bone and even the central nervous system (1,3).

The etiopathogenesis of morphea remains unclear, but it is thought to be due to a combination of genetic predisposition, which generates an autoimmune response triggered by factors such as trauma, radiation, drugs and infections (1,2). The vascular abnormalities are the result of the artery wall thickening, which reduces the lumen and alters vascularization in the area. Vascular dysfunction is one of the earliest changes observed, and may represent the initial event in the pathogenesis of the disease (1,2) 

The basis for the diagnosis of morphea is a histopathological assessment (1,4,5), which can vary in appearance depending on whether the injury is at an early or late stage (2). At the beginning of the disease, a perivascular lymphocytic infiltrate is observed with few plasma cells and eosinophils in the reticular dermis. Endothelial cells may be swollen and the collagen fibers thicker. During the more advanced stages of morphea, the inflammatory infiltrate disappears, and the collagen fibers become thicker, eosinophilic and huddled together. This increase in collagen is at the expense of the number of blood vessels and the size of the lumen (2,6). Morphea patients may present positive ANA autoantibodies in their blood. Antibody titers do not seem to be related to the course or severity of the disease (2,7,8).

To date, few cases of linear scleroderma with intraoral involvement have been reported (1,4,5,9). Below, we present the case of a 13-year-old girl diagnosed with severe intraoral linear scleroderma with involvement of the gingiva and underlying alveolar bone, besides a brief review of the literature.

## Case Report

A 13-year-old girl was referred to the diagnosis service of the Faculty of Dentistry of Universidad Andres Bello, Viña del Mar, Chile, because of increased mobility of her upper front incisors and the occurrence of a white spot on her upper lip. She previously attended a private clinic where they performed root canal treatments on the upper left lateral and central incisors. She reported no systemic diseases and no family history of similar diseases.

**Figure 1:**
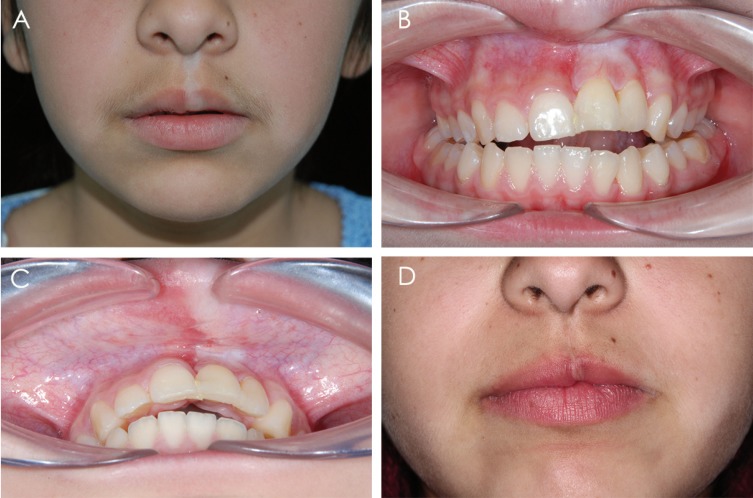
A) White hypopigmented line in the skin and vermilion of the upper lip. B) Commitment of the attached gingiva related to the upper left central and lateral incisors. C)Labial mucosa involvement. D) Appearance of the skin and vermilion of the upper lip after 2 years of treatment.

**Figure 2:**
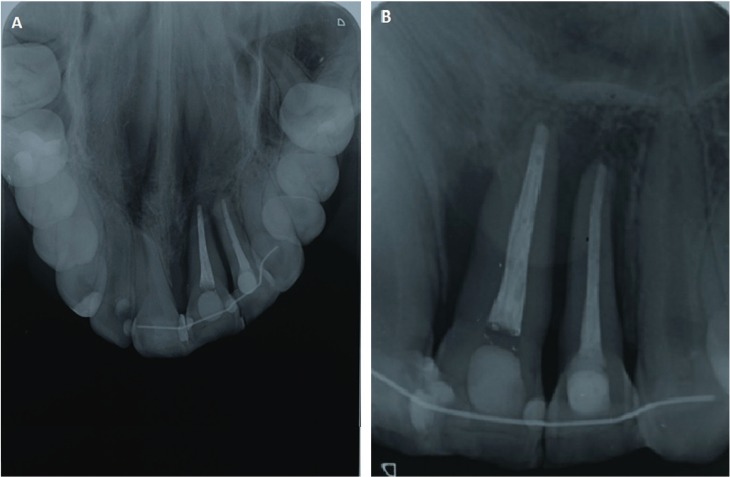
A) Occlusal Radiograph. B) Periapical radiograph with advanced vertical alveolar bone loss.

**Figure 3:**
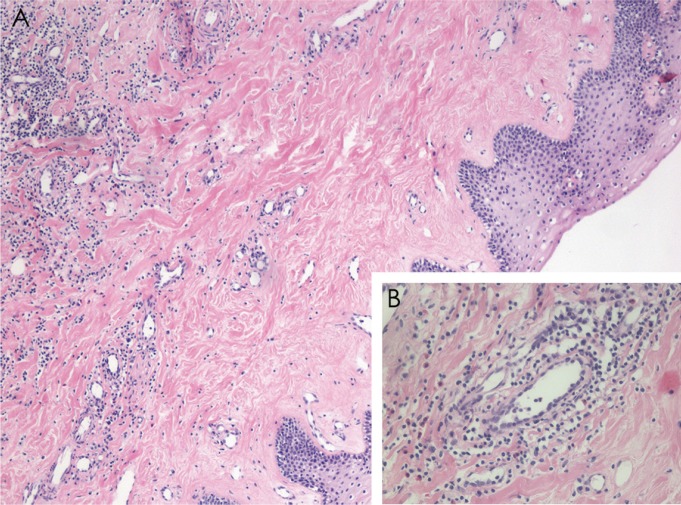
Histopathological findings. A) Biopsy of the labial mucosa under a light microscope, in which marked fibrosis of the lamina propria is observed, with hyalinization of collagen and a lymphocytic infiltrate with a perivascular and periductal distribution (hematoxylin-eosin staining, 4x). B) Higher magnification of an area with a perivascular mononuclear infiltrate (hematoxylin-eosin staining, 40x).

Clinical examination showed a hypopigmented white line on the skin of the left side of the upper lip, which continued through the vermilion and the lip mucosa, including the gingiva in relation to the upper left lateral and central incisors. Both teeth had a small gingival recession (Fig. 1 A-C), increased mobility and were slightly intruded, causing an anterior open bite. X-rays revealed advanced vertical alveolar bone loss (Fig. 2 A,B), while buccopalatal sections of computerized axial tomography (CAT) showed loss of the buccal alveolar ridge of both teeth. Linear white lesions of ≈1cm were also present on the dorsal and ventral surfaces of the tongue. An incisional biopsy of the affected labial mucosa was performed. Histopathological analysis of the area showed a marked thickening of collagen in the lamina propia, associated with a perivascular lymphomonocytic infiltrate and a strong presence of eosinophils. (Fig. 3 A,B).

The blood analysis, including a complete blood count, biochemical profile and serum levels of complement C3 and C4 were found normal. ENA, ANA and ANCA antibodies were negative. Echo-Doppler ultrasound showed the left facial and labial artery lumen diameters to be diminished by 50% compared with the right side arteries.

The patient was diagnosed with localized scleroderma (morphea). In collaboration with rheumatology consultants, an initial treatment of prednisone 15 mg/day, methotrexate (MTX) 20 mg/week (intramuscular) and folic acid 5 mg daily after the dose of MTX was given. The patient reported no adverse side effects.

The drug therapy described above was maintained for 15 months. No other lesions appeared during this period and the existing ones underwent no change or became smaller (Fig. 1D). The patient is currently (before the submission of this case) on a regular dose of MTX 2.5 mg/week, and 5mg/day of folic acid on the methotrexate-free days. No adverse side effects have been reported so far.

## Discussion

Localized scleroderma, is an inflammatory disease that primarily affects the skin and underlying tissues, leading to sclerosis of the affected tissues. Besides the genetic predisposition, there are environmental triggers (traumatic factors, radiation, medications and infections) (1,2). The disease may have an autoimmune basis due to the presence of positive ANA autoantibodies in 20-80% of patients (6). It usually affects young women between 4 -20-years-old, with a wide range of clinical presentations (5).

The typical presentation of morphea corresponds to a single linear injury with unilateral distribution that commonly affects the extremities, face and/or parietal region of the scalp (1,3,4), showing a linear zone of alopecia, with indurated skin and a shiny ivory color. It may or may not distort the underlying bone. Fortunately, in our case, the patient did no develop scalp lesions. The lesions may extend to the cheek, nose and/or upper lip, causing deformities or asymmetries of the facial structures or altering the implantation tissue of the teeth (3). Nevertheless, oral involvement in morphea is an uncommon feature. To our knowledge, there are only 20 cases reporting oral involvement (1,3-5,9). When the oral tissues are affected, it seems to have a predilection for the labial mucosa and upper anterior teeth as well as the soft palate and tongue. The most typical lesion is a white patch or a fibrotic area affecting the labial mucosa and/or gingiva causing gingival recession, loss of alveolar bone and tooth mobility (5). Van der Veken *et al.*, (1) reported a case of a 19-year-old women with morphea showing a pale linear lesion extending from her nose to her upper lip on the right-hand side of her face, with a progressive recession of the gingiva between the central and lateral incisors of the same side. The clinical features of this case are very similar to ours, but differ in the extension of the skin lesions and bone loss. In our case, the skin involvement was minimal and the bone loss was severe, while in this case, the cutaneous compromised was quite severe, but the bone involvement was just limited to a widening of the periodontal ligament on the mesial side of the central incisors.

Pace *et al.*, (4) reported in 2010 a case of frontal linear scleroderma with intraoral involvement. Althought the compromise of the skin was much more aggressive in this case, the intraoral involvement had some similiraties with ours. Similar to the present report, the authors reported the presence of a fibrotic lesion extending onto the attached gingiva, associated with a mobile upper right central incisor together with marked gingival recession on the mesial aspect of the lateral incisor.

Tang *et al.*, (5) reported the case of a 20-year-old female with a progressive recession of the gingiva around the premolars and molars on her left maxilla and a white plaque over the left upper vestibular mucosa and hard palate. The affected teeth showed bone resorption and a widened periodonal ligament. Histopathological features showed hialynization of subepithelial collagen and the presence of perivascular lymphocytic infiltrates. This characteristics are very similar to the ones present in our case.

Clinical, radiological and histopathological features of other reports of oral involvement in lineal scleroderma are very similar to the ones present in our case (1,3-5). Some differences could be seen regarding the extension of the gingival and alveolar bone compromise, probably attributed to the progression stage at the moment the diagnosis was made.

The main differential diagnoses for localized morphea is general morphea. General morphea presents with white lesions affecting two different anatomical regions. In the present case the lesions only affected the orofacial region. Because of the vasculitis ob-served in the histopathological analysis, Wegener´s granulomatosis has to be ruled out. In the present report, the patient had nega-tive ANCA antibodies and exhibit no clinical feature suggestive of Wegener´s granulomatosis.

There is still no standardized and effective treatment for linear scleroderma. Today, the most commonly used and accepted drugs for treatment are MTX and systemic glucocorticoids, used together most of the time (3,7,10-14). Zulian *et al.* concluded that long-term MTX therapy stops the progression of the disease and improves the appearance of clinical manifestations. Our patient took low doses of prednisone for 15 months, which was withdrawn stepwise, then maintained to date (two years after the start of treatment) only with oral MTX. With treatment, the progression of the disease stopped and the white line on the lip became sligh-tly less noticeable (Fig. 1D). Currently, the patient has not reported discomfort or adverse side effects to the drug therapy. The discrepancies in the management of reported cases may occur due to the fact that there are no standardized measurement instruments to compare the progress of patients before, during and after treatment, leading to great variability in the results of the reported studies in the literature. Finally, there are an insufficient number of controlled prospective studies that certify and evaluate the effectiveness of methotrexate (with or without corticosteroids) in the long term. The goal of the treatment was to stop progression of the morphea lesions.
